# Carbonized Aminal-Linked Porous Organic Polymers Containing Pyrene and Triazine Units for Gas Uptake and Energy Storage

**DOI:** 10.3390/polym15081891

**Published:** 2023-04-14

**Authors:** Aya Osama Mousa, Mohamed Gamal Mohamed, Cheng-Hsin Chuang, Shiao-Wei Kuo

**Affiliations:** 1 Department of Materials and Optoelectronic Science, Center of Crystal Research, National Sun Yat-sen University, Kaohsiung 804, Taiwan; 2 Institute of Medical Science and Technology, College of Medicine, National Sun Yat-sen University, Kaohsiung 804201, Taiwan; 3 Chemistry Department, Faculty of Science, Assiut University, Assiut 71515, Egypt; 4 Department of Medicinal and Applied Chemistry, Kaohsiung Medical University, Kaohsiung 807, Taiwan

**Keywords:** porous organic polymers, Schiff base condensation reaction, carbonization, gas uptake, electrochemical performance

## Abstract

Porous organic polymers (POPs) have plenteous exciting features due to their attractive combination of microporosity with π-conjugation. Nevertheless, electrodes based on their pristine forms suffer from severe poverty of electrical conductivity, precluding their employment within electrochemical appliances. The electrical conductivity of POPs may be significantly improved and their porosity properties could be further customized by direct carbonization. In this study, we successfully prepared a microporous carbon material (Py-PDT POP-600) by the carbonization of Py-PDT POP, which was designed using a condensation reaction between 6,6′-(1,4-phenylene)bis(1,3,5-triazine-2,4-diamine) (PDA-4NH_2_) and 4,4′,4′′,4′′′-(pyrene-1,3,6,8-tetrayl)tetrabenzaldehyde (Py-Ph-4CHO) in the presence of dimethyl sulfoxide (DMSO) as a solvent. The obtained Py-PDT POP-600 with a high nitrogen content had a high surface area (up to 314 m^2^ g^−1^), high pore volume, and good thermal stability based on N_2_ adsorption/desorption data and a thermogravimetric analysis (TGA). Owing to the good surface area, the as-prepared Py-PDT POP-600 showed excellent performance in CO_2_ uptake (2.7 mmol g^−1^ at 298 K) and a high specific capacitance of 550 F g^−1^ at 0.5 A g^−1^ compared with the pristine Py-PDT POP (0.24 mmol g^−1^ and 28 F g^−1^).

## 1. Introduction

Recently, the development of energy storage technologies to suit the modern needs of higher energy densities and specific powers is considered to be a hot global concern within academia and industrial fields [1,2,3,4,5,6,7,8,9,10]. Indeed, replacing fossil fuels via the use of batteries and supercapacitors can diminish severe global warming, in addition to being eco-friendly [11,12,13,14,15]. Ultra-capacitors or even electric double-layer capacitors are nominated supercapacitors; both discriminate over classical batteries by their long cycle life as well as their lightweight, low internal resistance, high power density, low servicing, reasonable energy density, flexibility, and wide thermal stabilities [16,17,18,19,20]. Due to its benefits, the supercapacitor has received much interest as a future energy storage technology. According to the differences in energy storage mechanisms, supercapacitors can be classified as redox electrochemical capacitors (pseudocapacitors), hybrid capacitors, and electrochemical double-layer capacitors (EDLCs) [21,22,23,24,25,26,27].

Conjugated microporous polymers (CMPs), covalent organic frameworks (COFs), polymers of intrinsic microporosity (PIMs), and hyper-cross-linked polymers (HCPs) have been considered as different types of porous organic polymers (POPs) [28,29,30,31,32,33,34,35]. POPs are an absorbent material that can be synthesized using many reactions such as the Schiff base reaction, Suzuki cross-coupling reaction, Sonogashira–Hagihara coupling reaction, Yamamoto coupling reaction, and Friedel–Crafts reaction [36,37,38,39,40,41,42,43,44,45,46]. POPs are particularly intriguing because of the unique characteristics of prolonged conjugation with persistent microporosity [40,41,42,43,44,45,46]. The electrical conductivity and pore structure of POPs might significantly improve with direct carbonization.

Currently, environmental problems have gained massive interest from researchers, specifically after the modern industrial revolution, and global warming is considered to be one of these hot problems [47,48,49,50]. The term “global warming” refers to the warming of the earth’s atmosphere and seas as a result of growing greenhouse gas concentrations brought on by human activities such as burning fossil fuels (coal, oil, and gas) and extensive deforestation [51,52,53]. From the beginning of the Industrial Revolution in 1750, carbon dioxide (CO_2_), one of the principal greenhouse gases linked to global climate change, has increased by more than 40%. There were significant environmental issues in May 2021 when atmospheric carbon dioxide levels hit a record high of 419 ppm [54,55]. Thus, reducing and controlling carbon emissions are pressing global concerns [56,57,58].

Porous organic polymers, metal–organic frameworks (MOFs), and activated carbons are frequently used for CO_2_ capture. POPs have become more popular and are considered to be possible porous material candidates that could effectively handle the carbon capture issue due to their simple synthesis and post-functionalization as well as excellent physiochemical stability and selectivity. POPs offer several benefits as a type of effective carbon capture material, including: (1) POPs are made of rigid monomers, which give rise to permanent porosity in the polymers; (2) there are numerous bond formation methods and cross-linking reactions, which give rise to polymers with different topological structures and tunable pore structures; (3) the cross-linking of lighter elements is used to create POPs, which results in polymers with high CO_2_ mass capacities; and (4) POPs are made of covalent bonds, giving rise to polymers with good physiochemical stability [35,36,37,38,39,40]. Thus, POPs with a specific performance and pore structure could be created to fit the demand for CO_2_ capture, energy storage, catalysis, and photocatalysis [34,38]. POPs have been used as a precursor for porous carbon materials (PCMs) [45]. PCMs derived from POPs have good properties such as large surface areas, high pore volumes, excellent electrical conductivity, and good thermal, mechanical, and chemical stabilities, so their preparation has attracted significant attention. Subsequently, porous carbonaceous materials have been widely applied in numerous real-life applications such as gas capture, dyes and iodine capture, fuel cells, electromagnetic interface shielding, catalysis, water treatment and purification, electrochemical energy storage in batteries and supercapacitors, and gas separation [40,41,42,43,44,45,46].

In this work, we prepared Py-PDT POP through a condensation reaction between 6,6′-(1,4-phenylene)bis(1,3,5-triazine-2,4-diamine) (PDA-4NH_2_) and 4,4′,4′′,4′′′-(pyrene-1,3,6,8-tetrayl)tetrabenzaldehyde (Py-Ph-4CHO). We then prepared a N-rich porous carbon material (Py-PDT POP-600), derived from the carbonization of Py-PDT POP at 600 °C. The properties, including the thermal degradation temperature, char yield, molecular structures, texture, porosity, and crystallinity of Py-PDT POP and Py-PDT POP-600, were investigated utilizing spectroscopic and microscopic techniques such as thermal gravimetric analysis (TGA), nuclear magnetic resonance (NMR), solid-state Fourier transform infrared (FTIR), Brunauer–Emmett–Teller (BET), transmission electron microscopy (TEM), and scanning electron microscopy (SEM). Furthermore, the electrochemical analysis was performed using cyclic voltammetry (CV) and galvanostatic charge−discharge (GCD) to investigate the impact of carbonization on the capacitive behavior of Py-PDT POP. Furthermore, CO_2_ uptake was measured to explore the potential application in gas capture. It was found that the carbonization process was an effective technique to enhance the porosity of Py-PDT POP, which is extremely helpful for the enhancement of the CO_2_ adsorption capacity and capacitive behavior.

## 2. Experimental Section

### 2.1. Materials

4-Formylphenylboronic acid (FP-BO), tetrakis(triphenylphosphine)palladium [Pd(PPh_3_)_4_], 2-cyanoguanidine, potassium carbonate (K_2_CO_3_), hydrochloric acid (HCl), dimethyl sulfoxide (DMSO), 1,4-dioxane (DO), 1,4-dicyanobenzene (BZ-2CN), dimethylformamide (DMF), anhydrous magnesium sulfate (MgSO_4_), and potassium hydroxide (KOH) were purchased from Alfa Aesar Sigma-Aldrich (Saint Louis, MO, USA). The 1,3,6,8-tetrabromopyrene (Py-Br_4_) monomer employed in this study was acquired using our reported methods (Scheme S1) [59].

### 2.2. Synthesis of 1,3,6,8-Tetrakis(4-formylphenyl)pyrene (Py-Ph-4CHO)

**Scheme 1:**
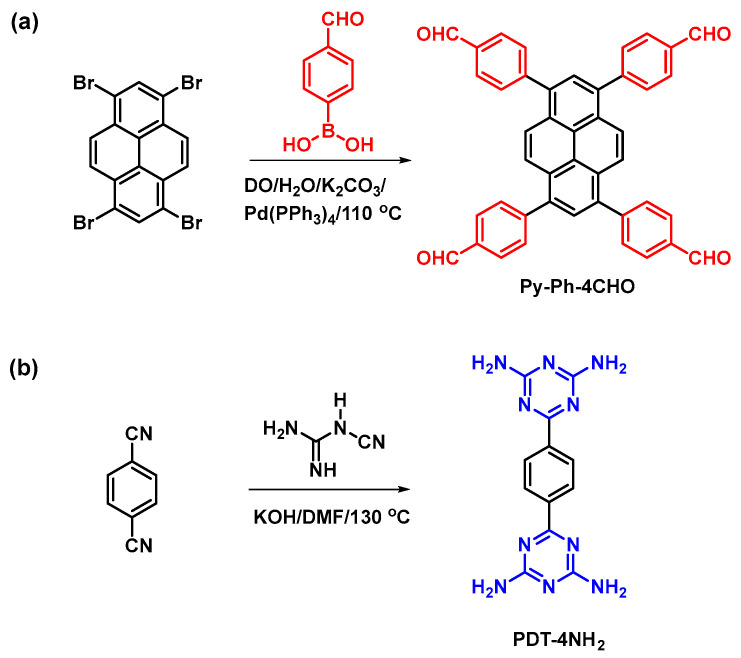
Synthesis of Py-Ph-4CHO (**a**) and PDT-4NH_2_ (**b**) from Py-Br_4_ and BZ-2CN.

**Figure 1:**
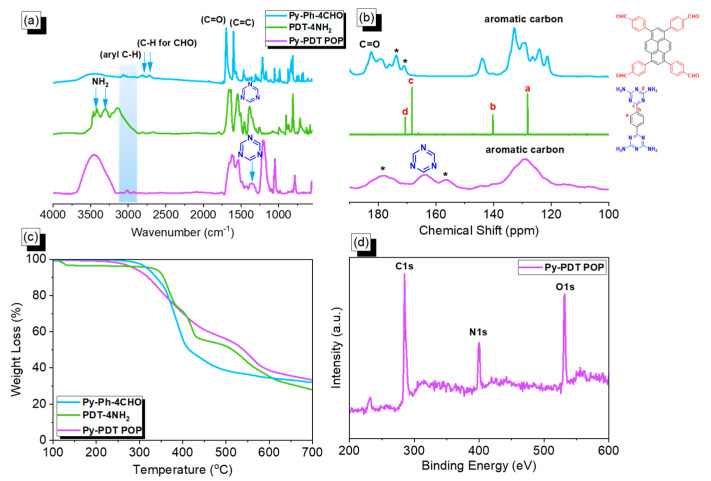
FTIR (**a**), ssNMR (**b**), and TGA (**c**) plots of Py-Ph-4CHO, PDT-4NH_2_, and Py-PDT POP, recorded at 25 °C; (**d**) XPS pattern of Py-PDT POP. * is the side band of solid-state nuclear magnetic resonance spectroscopy (NMR).

Py-Br_4_ (2.00 g, 3.86 mmol), FP-BO (23.2 mmol), K_2_CO_3_ (4.2 g, 30 mmol), and Pd(PPh_3_)_4_ (0.24 g, 0.2 mmol) in dry DO (60 mL)/H_2_O (20 mL) were added to a reaction flask. The flask was stirred under nitrogen and kept at 110 °C for three days. The obtained yellow suspension was discharged into a beaker containing H_2_O. After that, the precipitate was separated and exposed to washing processes via 2 M HCl (40 mL). The powder was extracted using CHCl_3_ (3 × 100 mL) and dried over MgSO_4_. Furthermore, the solvent was evaporated using a rotary evaporator and then recrystallized through hot CHCl_3_ to afford a pure solid powder with a bright yellow color (Scheme 1a; 1.7 g, 85%). The FTIR (KBr, cm^−1^; Figure 1a) were 3061, 1700, and 1598. The ^1^H NMR data of Py-Ph-4CHO is not provided in this study due to its poor solubility in all organic solvents. The ssNMR (ppm; Figure 1b): 183 (CHO) and 144–121 (aromatic rings).

### 2.3. Synthesis of 6,6′-(1,4-Phenylene)bis(1,3,5-triazine-2,4-diamine) (PDT-4NH_2_)

A mixture of KOH (1.124 g, 20 mmol) and 2-cyanoguanidine (4.048 g, 48 mmol) in DMF (160 mL) was added to a flask containing BZ-2CN (1.544 g, 9.2 mmol) in DMF (40 mL). The flask was magnetically stirred under nitrogen at 130 °C for 20 h (refluxing system). The obtained suspension was washed with MeOH and EtOH many times and dried to afford PDT-4NH_2_ as a white powder (Scheme 1b; 75%). The FTIR (KBr, cm^−1^; Figure 1a): 3300, 3123, and 1547. The ^13^C NMR (125 MHz, δ, ppm; Figure 1b): 170.65 (d), 168.46 (c), 139.96 (b), and 127.91 (a).

### 2.4. Synthesis of Py-PDT POP

**Scheme 2:**
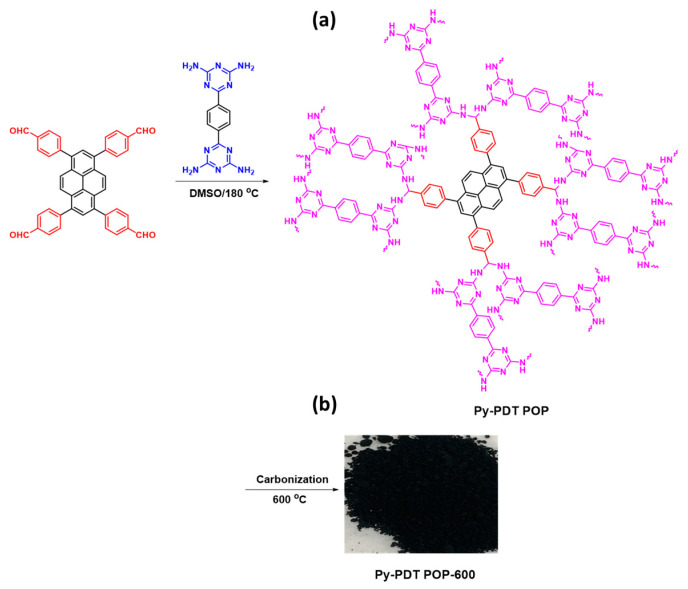
Synthesis of Py-PDT POP (**a**) and Py-PDT POP-600 (**b**) from Py-Ph-4CHO and PDT-4NH_2_.

PDT-4NH_2_ (0.26 g, 0.87 mmol), Py-Ph-4CHO (0.17 g, 0.27 mmol), and DMSO (20 mL) were added into a Schlenk flask. The flask was exposed to a thaw cycle three times. The flask was then heated to 180 °C and stirred for three days under nitrogen. After cooling the flask to room temperature, the product was separated by filtration and washed with DMF, MeOH, and acetone. The brown powder of Py-PDT POP was dried under a vacuum at 100 °C for 24 h. Finally, Py-PDT POP was obtained as a dark brown powder (70%; Scheme 2a).

### 2.5. Synthesis of Py-PDT POP-600

The as-prepared Py-PDT POP was placed in a ceramic boat into a tubular furnace and carbonized at 600 °C for 8 h (heating rate of 5 °C min^−1^) under a N_2_ atmosphere. After allowing the tube furnace’s temperature to reach the ambient temperature, the carbonized product was collected as a black powder and named Py-PDT POP-600 (Scheme 2b).

## 3. Results and Discussion

### 3.1. Synthesis and Characterization of Py-Ph-4CHO, PDT-4NH_2_, and Py-PDT

Scheme 1 shows the synthesis of the Py-Ph-4CHO and PDT-4NH_2_ monomers. A pyrene molecule was reacted with a neat bromine solution in the presence of C_6_H_5_NO_2_ at an elevated temperature (120 °C) to afford Py-Br_4_ as a light green solid with a high yield (Scheme S1). The obtained Py-Br_4_ was insoluble in all organic solvents and used in the next step without purification. The bands in the FTIR pattern of Py-Br_4_ centered at 3053 and 682 cm^−1^ for aromatic C-H and C-Br units (Figure S1). The Py-Ph-4CHO monomer was synthesized through the Suzuki coupling reaction of Py-Br_4_ with FP-BO in the presence of K_2_CO_3_/DO/H_2_O at 110 °C for three days to afford a yellow solid (Scheme 1a). The ^1^H NMR results of the Py-Br_4_ and Py-Ph-4CHO monomers are not provided because of their poor solubility. A reaction of 2-cyanoguanidine with 1,4-dicyanobenzene (BZ-2CN) was then created in the presence of KOH and DMF to obtain PDT-4NH_2_ as a white powder (Scheme 1b). The proton’s signals appeared at 6.8 and 8.3 ppm due to the presence of an amino group and a phenyl ring in the PDT-4NH_2_ (Figure S2). Scheme 2a illustrates the synthetic route for preparing the porous organic polymer named Py-PDT POP from PDT-4NH_2_ and Py-Ph-4CHO as building monomers. The Py-PDT POP was constructed through a Schiff base polycondensation reaction between 6,6′-(1,4-phenylene)bis(1,3,5-triazine-2,4-diamine) (PDA-4NH_2_) and 4,4′,4′′,4′′′-(pyrene-1,3,6,8-tetrayl)tetrabenzaldehyde (Py-Ph-4CHO) in the presence of DMSO at 180 °C for 72 h under N_2_ without using any catalyst (Scheme 2a). The as-synthesized Py-PDT POP was washed with DMF, DMSO, THF, MeOH, and acetone to remove the unreacted Py-Ph-4CHO and PDT-4NH_2_. The Py-PDT POP was then placed into a tube furnace for calcination at 600 °C under N_2_ for 8 h to afford Py-PDT POP-600 as a black precipitate (Scheme 2b). Several instrumental techniques (FTIR, ssNMR, TGA, TEM, SEM, BET, and XPS) were used to characterize our porous Py-PDT POP and Py-PDT POP-600 materials.

The chemical molecular structure of building monomers (Py-Ph-4CHO and PDT-4NH_2_) and the obtained Py-PDT POP were confirmed using solid-state ^13^C NMR and FTIR, as presented in Figure 1. Figure 1a displays the FTIR profile (recorded at 25 °C) of Py-Ph-4CHO, PDT-4NH_2_, and Py-PDT POP. The FTIR spectrum of Py-Ph-4CHO displayed an absorption band at 3061 cm^−1^ for the C-H aromatic, 2810 and 2717 cm^−1^ for the aldehydic C-H, 1700 cm^−1^ for C=O, and 1598 cm^−1^ for the C=C bond. The FTIR spectrum of PDT-4NH_2_ showed absorption bands at 3300, 3123, and 1624 for the NH_2_ group, aromatic C-H, and C=C bonds. The peaks at ca. 1547 and 1364 cm^−1^ in the FTIR spectra (Figure 1a) of PDT-4NH_2_ and Py-PDT POP indicated the triazine moiety’s existence in the chemical structure [60,61]. Comparing the FTIR spectrum of the Py-Ph-4CHO monomer and the as-prepared Py-PDT POP revealed that the characteristic absorption peak intensity of the aldehydic units became weak in the FTIR profile of Py-PDT POP, indicating a complete condensation reaction between Py-Ph-4CHO and PDT-4NH_2_ to afford Py-PDT POP with a high cross-linking density and aminal linkage. The chemical structures of PDT-4NH_2_, Py-Ph-4CHO, and Py-PDT POP were further examined by solid-state ^13^C NMR measurements (recorded at 25 °C; Figure 1b). The carbon’s signals in the ssNMR of Py-Ph-4CHO centered at 183 ppm and in the range of 144–121 ppm corresponded with the C=O of the aldehydic group and aromatic rings (Figure 1b). The carbon peaks of PDT-4NH_2_ were observed at 168.46 ppm due to the C=N unit in the triazine ring and signals at 139.96 and 127.91 ppm were attributed to aromatic carbons (Figure 1b). The ssNMR profile displayed peaks at 164 and 129 ppm in Py-PDT POP due to the presence of carbon atoms for the C=N units in the triazine ring and aromatic carbons, respectively (Figure 1b). To evaluate the thermal stability of our materials (PDT-4NH_2_, Py-Ph-4CHO, and Py-PDT POP), we performed TGA under a N_2_ stream between temperatures of 40 and 800 °C (Figure 1c). The TGA results revealed that the 10% weight loss values of PDT-4NH_2_, Py-Ph-4CHO, and Py-PDT POP were 355, 338, and 320 °C, respectively. The char yield estimations at 800 °C for PDT-4NH_2_, Py-Ph-4CHO, and Py-PDT POP were 35, 34, and 39 wt%, respectively. Furthermore, the presence of nitrogen, oxygen, and carbon atoms on the surface of Py-PDT POP was confirmed using an XPS analysis, as displayed in Figure 1d. The XPS profile of Py-PDT POP showed signals at 284.5 eV, 400.17 eV, and 531.39 eV, which were attributed to the C atoms of the aromatic rings, N atoms in the triazine units, and O atoms for the terminal CHO group, respectively. According to the FTIR and TGA results, the information mentioned above supported the formation of the aminal linkage to construct the Py-PDT POP framework with good thermal stability. El-Kadri et al. prepared fluorescent NRAPOP-1 and NRAPOP-2 through aminal linkage for I_2_ capture and Fe^3+^ detection [62]. The same group constructed TALPOP based on anthracene and triazine units for I_2_ uptake [63].

**Figure 2:**
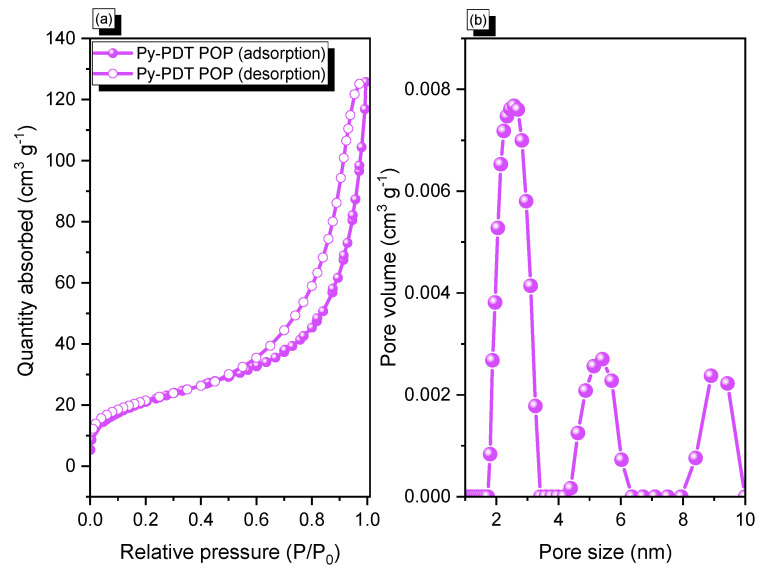
N_2_ adsorption/desorption (**a**) and pore size (PSD) curves (**b**) of Py-PDT POP.

The BET surface area, pore size diameter, and total pore volume of Py-PDT POP before the carbonization process were investigated by N_2_ adsorption/desorption measurements at 77 K (Figure 2). The N_2_ adsorption isotherm of Py-PDT POP exhibited minimal N_2_ uptake at low pressures. It rapidly increased at high pressures, indicating that Py-PDT POP could be classified as type IV, according to the IUPAC classification. This suggested the presence of mesopores in the Py-PDT POP framework, as shown in Figure 2a. Moreover, the value of the BET surface area of Py-PDT POP was calculated from the N_2_ adsorption/desorption isotherm, which was 76 m^2^ g^−1^, with a total pore volume of 0.2 cm^3^ g^−1^. The nonlocal density functional theory (NLDFT) was used to determine the pore diameters from their sorption isotherms. The pore size profile of Py-PDT POP peaked at 2.5, 5.4, and 8.8 nm, indicating that Py-PDT POP contained mesopore structures, based on the pore size (Figure 2b).

**Figure 3:**
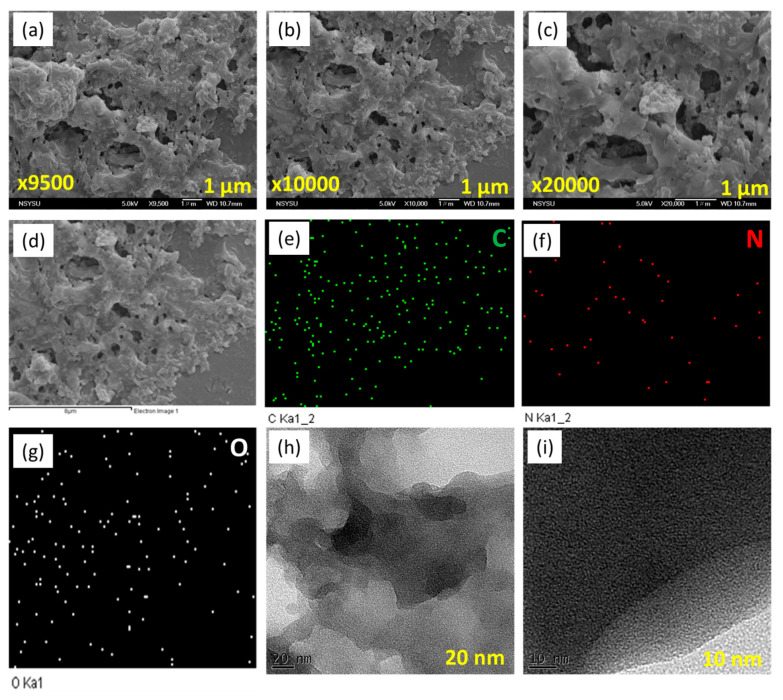
SEM (**a**–**c**), EDS-SEM (**d**–**g**), and TEM (**h**,**i**) images of Py-PDT POP.

Moreover, we examined the morphology of Py-PDT POP using high-resolution transmission electron microscopy (HR-TEM) and field emission scanning electron microscopy (FE-SEM). Py-PDT POP contained aggregated particles with pores, based on the FE-SEM imaging (Figure 3a–c). SEM-EDS (energy-dispersive X-ray scattering) was used to confirm the compositions and different elements in the chemical structures of Py-PDT POP. Figure 3d–g show evidence of carbon, nitrogen, and oxygen atoms distributed in the Py-PDT POP skeleton. The HR-TEM images (Figure 3h,i) showed the existence of bright and alternating dark patches, which likely suggested that Py-PDT POP included porous networks.

### 3.2. Porosity, Thermal Stability, and Morphology of Py-PDT POP-600

**Figure 4:**
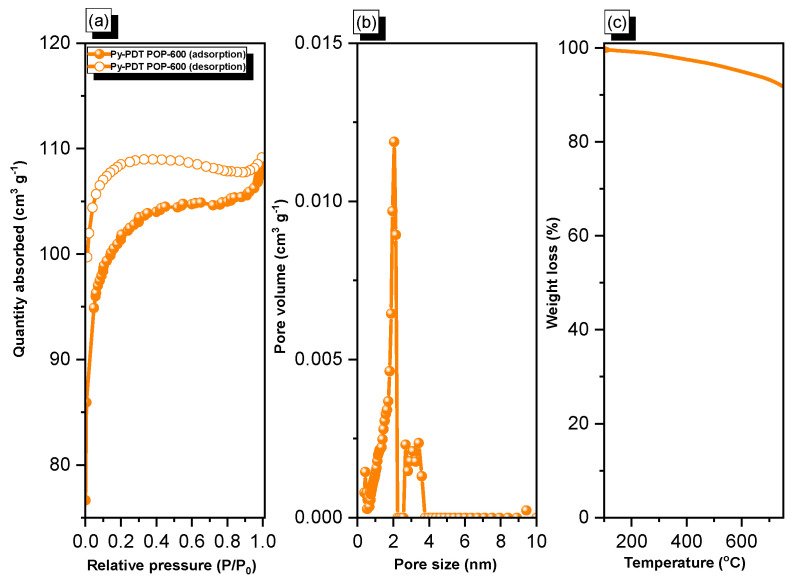
N_2_ adsorption/desorption (**a**), pore size (PSD) (**b**), and TGA (**c**) curves of Py-PDT POP-600.

As shown in Figure 4a, Py-PDT POP-600 exhibited a fast N_2_ capture ability at low pressures, indicating micropores in the material. Furthermore, it continued to increase for N_2_ adsorption at high pressures with a hysteresis loop, suggesting the presence of mesopores in this material. Based on the IUPAC nomenclature, the adsorption/desorption isotherm of Py-PDT POP-600 possessed both types I and type IV. The BET surface area of Py-PDT POP-600 was calculated to be 314 m^2^ g^−1^. The pore size distribution (PSD) of Py-PDT POP-600 was determined by applying the nonlocal density functional theory (NLDFT). The pore size distribution (PSD) curve (Figure 4b) showed that Py-PDT POP-600 possessed both micropores and mesopores (average diameters of 1.9 and 2.7 nm, respectively). Compared with the precursor Py-PDT POP, the porosity of Py-PDT POP-600 was considerably enhanced. Furthermore, we used TGA to examine the thermal stability of Py-PDT POP-600 (Figure 4c). The degradation temperature of Py-PDT POP-600 after losing 10% of its original weight was 769 °C. Moreover, the char yield for Py-PDT POP-600 was 90 wt%. The TGA results implied the outstanding thermal stability of Py-PDT POP-600; this was also attributed to the carbonization process of the as-prepared Py-PDT POP, which granted our materials sheet-like structures and, consequently, a higher stacking effect between the layers.

**Figure 5:**
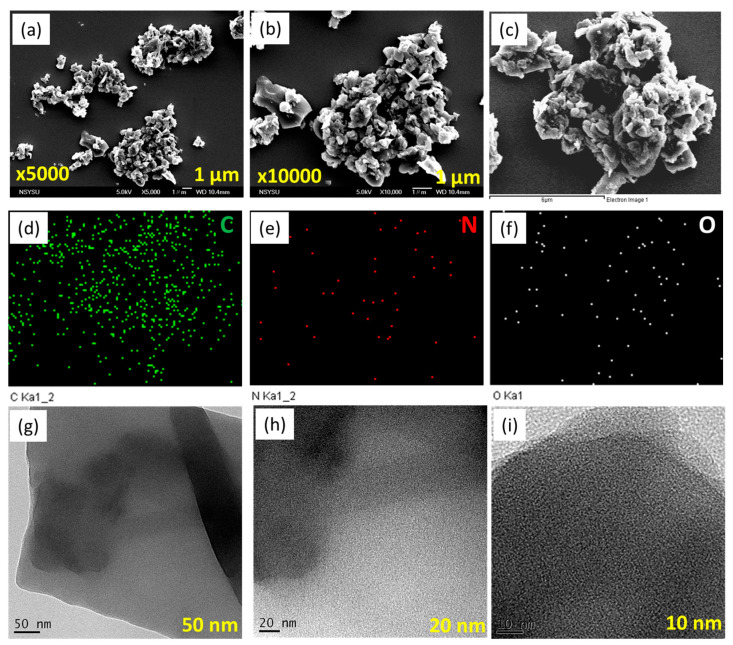
SEM (**a**,**b**), EDS-SEM (**c–f**), and TEM (**g**–**i**) images of Py-PDT POP-600.

SEM and TEM analyses were used to examine the morphology of our porous Py-PDT POP-600. The SEM images of Py-PDT POP-600 revealed an aggregation and sheet structure (Figure 5a,b). SEM-EDS was used to confirm the compositions and different elements in the chemical forms of Py-PDT POP-600. The data showed the presence of C, N, and O atoms distributed in the Py-PDT POP-600 skeleton (Figure 5c–f). Furthermore, the TEM images of Py-PDT POP-600 elucidated the rod-like and microporous structures (Figure 5g–i). As expected, the amorphous forms of both Py-PDT POP and Py-PDT POP-600 were revealed through the XRD analysis.

### 3.3. CO_2_ Uptake Performance for Py-PDT POP and Py-PDT POP-600 at 298 K

**Figure 6:**
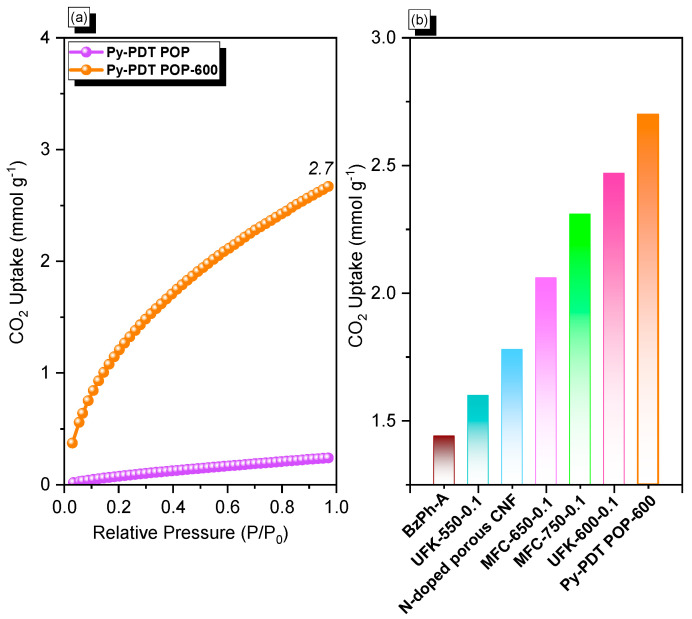
CO_2_ capacity (**a**) of Py-PDT POP and Py-PDT POP-600 and their performance compared with other materials (**b**).

Global warming is one of the severe consequences of industrial revolutions, so researchers continue to strive to find suitable solutions to minimize these environmental issues. As previously reported, the carbonization process at a higher temperature under N_2_ gas could enhance the CO_2_ uptake performance of the POP materials. As a result, we performed the calcination process for Py-PDT POP at 600 °C for 8 h to produce a black solid (Py-PDT POP-600), as indicated in Scheme 2b. According to the BET results, the resulting Py-PDT POP-600 material showed a larger pore volume, a higher surface area, and microporous characters compared with the pristine Py-PDT POP precursor. The CO_2_ isotherm measurements determined the CO_2_ uptake performance of Py-PDT POP and Py-PDT POP-600 at 298 K (Figure 6a). Py-PDT POP showed a low CO_2_ uptake of 0.24 mmol g^−1^. On the other hand, Py-PDT POP-600 showed an improvement in CO_2_ uptake. As expected, Py-PDT POP-600, with the highest BET surface area, offered the most increased CO_2_ uptake of 2.7 mmol g^−1^. As presented in Figure 6b, the CO_2_ capacity of Py-PDT POP-600 (2.7 mmol g^−1^) was higher than that of BZPh-A (1.44 mmol g^−1^) [64], UFK-550-0.1 (1.6 mmol g^−1^) [65], N-doped porous CNF (1.78 mmol g^−1^) [66], MFC-650-0.1(2.06 mmol g^−1^) [67], MFC-750-0.1 (2.31 mmol g^−1^) [67], and UFK-550-0.1 (2.47 mmol g^−1^) [65].

### 3.4. Electrochemical Performance of Py-PDT POP and Py-PDT POP-600

**Figure 7:**
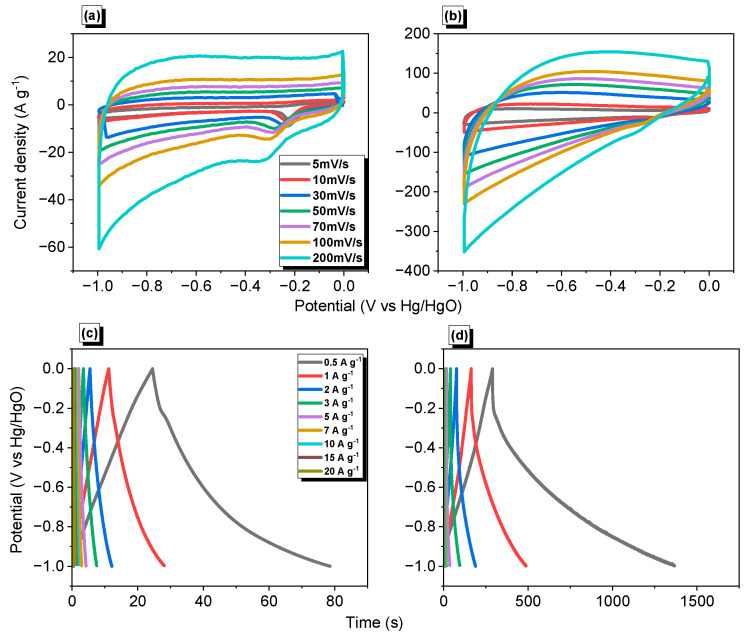
CV (**a**,**b**) and GCD (**c**,**d**) profiles of Py-PDT POP (**a**,**c**) and Py-PDT POP-600 (**b**,**d**).

As mentioned above, supercapacitor-based electrodes are challengeable nowadays; these devices also consider a green energy storage methodology. Encouraged by our synthesized material’s physical and chemical features and carbonized form, we investigated their electro- and capacitance behaviors. The electrochemical performances of our synthesized Py-PDT POP and Py-PDT POP-600 were estimated using cyclic voltammetry (CV) and galvanostatic charge−discharge (GCD) measurements, based on a three-electrode system incorporating glassy carbon, a platinum electrode, and Hg/HgO as the working, counter, and reference electrodes, respectively (Figure 7). The CV plateaus of Py-PDT POP represented numerous scans between 5 and 200 mV s^−1^ within a potential window of −1.0 to 0.0 V relative to Hg/HgO as a reference electrode. As shown in Figure 7a, Py-PDT POP could derive quasi-rectangular CV shapes in addition to palpable humbling and harmony, demonstrating its steady terms of the current sweep and revealing its capacitive feature to EDLC [41,42,61,68]. The CV plot of Py-PDT POP at a higher scan rate implied a symmetrical quasi-rectangular shape, elucidating its EDLC nature. Conversely, Py-PDT POP-600 (Figure 7b) showed a superior integrated rate, corresponding with a higher former capacitance than pristine POP. This result was attributed to the poor electrical conductivity of the pristine Py-PDT POP. The GCD measurements of Py-PDT POP and Py-PDT POP-600 at different current densities were investigated to evaluate their electrical capacitance performance. As emphasized by Figure 7c,d, the GCD plots of Py-PDT POP and Py-PDT POP-600 at various current densities implied a semi-triangular shape, consequently revealing the EDLC mechanism within their energy storage.

**Figure 8:**
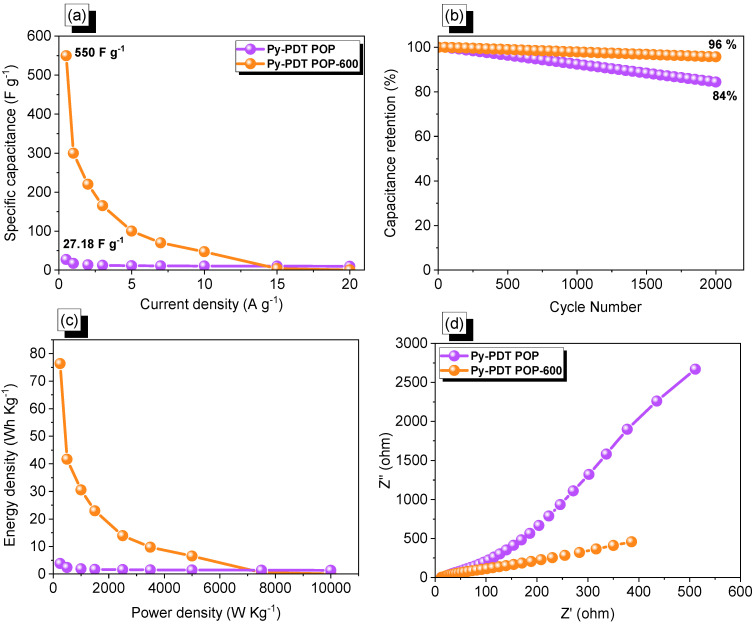
Capacitance (**a**) and cycling stability (**b**), Ragone (**c**) and EIS (**d**) profiles of Py-PDT POP and Py-PDT POP-600.

As we expected, the specific capacitance of Py-PDT POP-600 at a current density of 0.5 A g^−1^ was 550 F g^−1^, which was considered to be much higher than Py-PDT POP (28 F g^−1^ at 0.5 A g^−1^) (Figure 8a). We compared our Py-PDT POP-600 with other carbon porous materials and their derivatives, such as carbons derived from peach gum, hollow carbon–MoS_2_ carbon nanoplates, lignin-based and cellulose hydrogels, carbon composite and replicas obtained from a hybrid layered double hydroxide active container, tannic acid (TA), and carbon nanotubes (CNTs), and others [61,69,70,71,72,73,74,75,76,77,78,79,80]. The electrochemical performance of our Py-PDT POP-600 displayed an excellent electrochemical character (Table S1) [61,69,70,71,72,73,74,75,76,77,78,79,80]. The superb performance of Py-PDT POP-600 in energy storage applications was due to its high N content, surface area, pore volume, pore size, and prolonged conjugated structure [61,68]. The long-term stability of Py-PDT POP and Py-PDT POP-600 was investigated through cycling processes for 2000 cycles at a current density of 10 A g^−1^. As shown in Figure 8b, both Py-PDT POP and Py-PDT POP-600 showed capacitance retention of 84 and 96%, respectively. Accordingly, the Ragone plots of our materials (Figure 8c) emphasized that Py-PDT POP-600 possessed a maximum energy density of 76.38 Wh Kg^−1^, which was higher than pristine Py-PDT POP, which was 3.77 Wh Kg^−1^. Electrochemical impedance spectroscopy (EIS) investigations of the Py-PDT POP- and Py-PDT POP-600-based electrodes helped us to emphasize their kinetic behaviors. Nyquist graphs of the Py-PDT POP and Py-PDT POP-600 precursors implied small semi-circles at higher frequencies and a semi-straight line at lower frequencies (Figure 8d). The latter represented a lower resistance than the former, which revealed the lower resistance of Py-PDT POP-600. The lower resistance of Py-PDT POP-600 may have been due to the higher offering surface area of the electrode, consequently improving the surface wettability; hence, this enhanced the access of electrolyte ions to the current electrode.

## 4. Conclusions

In summary, Py-PDT POP was constructed and designed by reacting Py-Ph-4CHO with PDT-4NH_2_ in DMSO at 180 °C (free metal Schiff base condensation reaction). The molecular structure and thermal stability of the building units (Py-Ph-4CHO with PDT-4NH_2_) and the Py-PDT POP framework were carefully investigated through ssNMR, FTIR, and XPS measurements. The porosity property of Py-PDT POP was successfully enhanced through a carbonization approach at 600 °C for 8 h to access Py-PDT POP-600 as a black solid with a high surface area (314 m^2^ g^−1^), high T*_d10_* (769 °C), and high carbon residue (90 wt%), based on BET and TGA results. For the CO_2_ uptake and supercapacitor applications, the as-prepared Py-PDT POP-600 showed excellent performance in CO_2_ uptake (2.7 mmol g^−1^ at 298 K), a high specific capacitance (550 F g^−1^ at 0.5 A g^−1^), and retention stability (96%) compared with the Py-PDT POP framework. Therefore, the carbonization process improved the pore structure and significantly increased the POP electrochemical performance and CO_2_ capture. The obtained materials and findings presented here indicated that the multifunctional Py-PDT POP-600 precursor is an excellent candidate for gas adsorption and energy storage. Creating porous Py-PDT POP-600 by linking heteroatom-rich building units may open the door to creating innovative materials for various applications, including dyes and iodine absorption.

## Data Availability

The data presented in this study are available on request from the corresponding author.

## Supplementary Materials

The following supporting information can be downloaded at: https://www.mdpi.com/article/10.3390/polym15081891/s1, Scheme S1: Synthesis of Py-Br_4_; Figure S1: FTIR spectrum of Py-Br_4_; Figure S2: ^1^H NMR profile of PDT-4NH_2_; Table S1: Comparison between the capacity values of Py-PDT POP-600 with different reported data of three-electrode supercapacitor materials [61,69,70,71,72,73,74,75,76,77,78,79,80].

## References

[1] Xiong S., Liu J., Wang Y., Wang X., Chu J., Zhang R., Gong M., Wu B. (2022). Solvothermal synthesis of triphenylamine-based covalent organic framework nanofibers with excellent cycle stability for supercapacitor electrodes. J. Appl. Polym. Sci..

[2] Ejaz M., Mohamed M.G., Sharma S.U., Lee J.-T., Huang C.-F., Chen T., Kuo S.-W. (2022). An ultrastable porous polyhedral oligomeric silsesquioxane/tetraphenylthiophene hybrid as a high-performance electrode for supercapacitors. Molecules.

[3] Mondloch J.E., Bury W., Jimenez D.F., Kwon S., DeMarco E.J., Weston M.H., Amy A., Sarjeant A.A., Nguyen S.T., Stair P.C. (2013). Vapor-Phase Metalation by Atomic Layer Deposition in a Metal–Organic Framework. J. Am. Chem. Soc..

[4] Rafik F., Gualous H., Gallay R., Crausaz A., Berthon A. (2007). Frequency, thermal and voltage supercapacitor characterization and modeling. J. Power Sources.

[5] del Valle M.A., Gacitúa M.A., Hernández F., Luengo M., Hernández L.A. (2023). Nanostructured Conducting Polymers and Their Applications in Energy Storage Devices. Polymers.

[6] Chen D., Jiang K., Huang T., Shen G. (2020). Recent advances in fiber supercapacitors: Materials, device configurations, and applications. Adv. Mater..

[7] Samy M.M., Mohamed M.G., Sharma S.U., Chaganti S.V., Lee J.-T., Kuo S.-W. (2023). An Ultrastable Tetrabenzonaphthalene-Linked conjugated microporous polymer functioning as a high-performance electrode for supercapacitors. J. Taiwan Inst. Chem. Eng..

[8] Loganathan N.N., Perumal V., Pandian B.R., Atchudan R., Edison T.N.J.I., Ovinis M. (2022). Recent studies on polymeric materials for supercapacitor development. J. Energy Storage.

[9] Dehghani-Sanij A.R., Tharumalingam E., Dusseault M.B., Fraser R. (2019). Study of Energy Storage Systems and Environmental Challenges of Batteries. Renew. Sustain. Energy Rev..

[10] Mohamed M.G., Sharma S.U., Liu N.-Y., Mansoure T.H., Samy M.M., Chaganti S.V., Chang Y.-L., Lee J.-T., Kuo S.-W. (2022). Ultrastable covalent triazine organic framework based on anthracene moiety as platform for high-performance carbon dioxide adsorption and supercapacitors. Int. J. Mol. Sci..

[11] Zheng S., Li Q., Xue H., Pang H., Xu Q. (2020). A highly alkaline-stable metal oxide@ metal–organic framework composite for high-performance electrochemical energy storage. Natl. Sci..

[12] Mohamed M.G., Mansoure T.H., Samy M.M., Takashi Y., Mohammed A.A., Ahamad T., Alshehri S.M., Kim J., Matsagar B.M., Wu K.C.-W. (2022). Ultrastable Conjugated Microporous Polymers Containing Benzobisthiadiazole and Pyrene Building Blocks for Energy Storage Applications. Molecules.

[13] Shaikh N.S., Ubale S.B., Mane V.J., Shaikh J.S., Lokhande V.C., Praserthdam S., Lokhande C.D., Kanjanaboos P. (2022). Novel electrodes for supercapacitor: Conducting polymers, metal oxides, chalcogenides, carbides, nitrides, MXenes, and their composites with graphene. J. Alloys Compd..

[14] Zheng S., Sun Y., Xue H., Braunstein P., Huang W., Pang H. (2022). Dual-ligand and hard-soft-acid-base strategies to optimize metal-organic framework nanocrystals for stable electrochemical cycling performance. Natl. Sci..

[15] Tomboc G.M., Kim J., Wang Y., Son Y., Li J., Kim J.Y., Lee K. (2021). Hybrid layered double hydroxides as multifunctional nanomaterials for overall water splitting and supercapacitor applications. J. Mater. Chem. A.

[16] Shan X., Guo Z., Qu Z., Zou Y., Zhao L., Chen P. (2022). Boosting the performance of nickel–cobalt LDH cathode with phosphorus and selenium co-doping for hybrid supercapacitor. Mater. Res. Lett..

[17] Zaw N.Y.W., Jo S., Park J., Kitchamsetti N., Jayababu N., Kim D. (2022). Clay-assisted hierarchical growth of metal-telluride nanostructures as an anode material for hybrid supercapacitors. Appl. Clay Sci..

[18] Kitchamsetti N., Samtham M., Didwal P.N., Kumar D., Singh D., Bimli S., Chikate P.R., Basha D.A., Kumar S., Park C.-J. (2022). Theory abide experimental investigations on morphology driven enhancement of electrochemical energy storage performance for manganese titanate perovskites electrodes. J. Power Sources.

[19] Kitchamsetti N., Ma Y.-R., Shirage P.M., Devan R.S. (2020). Mesoporous perovskite of interlocked nickel titanate nanoparticles for efficient electrochemical supercapacitor electrode. J. Alloys Compd..

[20] Zhang Y., Gao X., Zhang Y., Gui J., Sun C., Zheng H., Guo S. (2022). High-efficiency self-charging power systems based on performance-enhanced hybrid nanogenerators and asymmetric supercapacitors for outdoor search and rescue. Nano Energy.

[21] Du W., Wang X., Zhan J., Sun X., Kang L., Jiang F., Zhang X., Shao Q., Dong M., Liu H. (2019). Biological cell template synthesis of nitrogen-doped porous hollow carbon spheres/MnO_2_ composites for high-performance asymmetric supercapacitors. Electrochim. Acta.

[22] Zhao D., Wang H., Bai Y., Yang H., Song H., Li B. (2022). Preparation of Advanced Multi-Porous Carbon Nanofibers for High-Performance Capacitive Electrodes in Supercapacitors. Polymers.

[23] Sahoo S., Kumar R., Joanni E., Singh R.K., Shim J.-J. (2022). Advances in pseudocapacitive and battery-like electrode materials for high performance supercapacitors. J. Mater. Chem. A.

[24] Han C., Tong J., Tang X., Zhou D., Duan H., Li B., Wang G. (2020). Boost anion storage capacity using conductive polymer as a pseudocapacitive cathode for high-energy and flexible lithium ion capacitors. ACS Appl. Mater. Interfaces.

[25] Ahmad Z., Kim W.-B., Kumar S., Yoon T.-H., Shim J.-J., Lee J.-S. (2022). Redox-active supercapacitor electrode from two-monomer-connected precursor (Pyrrole: Anthraquinonedisulfonic acid: Pyrrole) and sulfonated multi-walled carbon nanotube. Electrochim. Acta.

[26] Liu S., Kang L., Zhang J., Jun S.C., Yamauchi Y. (2021). Carbonaceous anode materials for non-aqueous sodium-and potassium-ion hybrid capacitors. ACS Energy Lett..

[27] Liu S., Kang L., Hu J., Jung E., Zhang J., Jun S.C., Yamauchi Y. (2021). Unlocking the potential of oxygen-deficient copper-doped Co_3_O_4_ nanocrystals confined in carbon as an advanced electrode for flexible solid-state supercapacitors. ACS Energy Lett..

[28] Mohamed M.G., Elsayed M.H., Ye Y., Samy M.M., Hassan A.E., Mansoure T.H., Wen Z., Chou H.-H., Chen K.-H., Kuo S.-W. (2023). Construction of Porous Organic/Inorganic Hybrid Polymers Based on Polyhedral Oligomeric Silsesquioxane for Energy Storage and Hydrogen Production from Water. Polymers.

[29] Liu T., Liu G. (2020). Porous Organic Materials Offer Vast Future Opportunities. Nat. Commun..

[30] Yuan R., Zhang M., Sun H. (2023). Design and Construction of an Azo-Functionalized POP for Reversibly Stimuli-Responsive CO_2_ Adsorption. Polymers.

[31] Panić B., Frey T., Borovina M., Konopka K., Sambolec M., Kodrin I., Biljan I. (2023). Synthesis and characterization of benzene- and triazine-based azo-bridged porous organic polymers. Polymers.

[32] Zhao X., Qi Y., Li J., Ma Q. (2022). Porous organic polymers derived from ferrocene and tetrahedral silicon-centered monomers for carbon dioxide sorption. Polymers.

[33] Cousins K., Zhang R. (2019). Highly porous organic polymers for hydrogen fuel storage. Polymers.

[34] Daliran S., Oveisi A.R., Peng Y., López-Magano A., Khajeh M., Mas-Ballesté R., Alemán J., Luque R., Garcia H. (2022). Metal–organic framework (MOF)-, covalent-organic framework (COF)-, and porous-organic polymers (POP)-catalyzed selective C–H bond activation and functionalization reactions. Chem. Soc. Rev..

[35] Singh N., Son S., An J., Kim I., Choi M., Kong N., Tao W., Kim J.S. (2021). Nanoscale porous organic polymers for drug delivery and advanced cancer theranostics. Chem. Soc. Rev..

[36] Amin K., Ashraf N., Mao L., Faul C.F., Wei Z. (2021). Conjugated microporous polymers for energy storage: Recent progress and challenges. Nano Energy.

[37] Lu Q., Wang X., Cao J., Chen C., Chen K., Zhao Z., Niu Z., Chen J. (2017). Freestanding carbon fiber cloth/sulfur composites for flexible room-temperature sodium-sulfur batteries. Energy Storage Mater..

[38] Magano A.L., Daliran S., Oveisi A.R., Mas-Ballesté R., Dhakshinamoorthy A., Alemán J., Garcia H., Luque R. (2022). Recent advances in the use of covalent organic frameworks as heterogeneous photocatalysts in organic synthesis. Adv. Mater..

[39] Young C., Park T., Yi J.W., Kim J., Hossain M.S.A., Kaneti Y.V., Yamauchi Y. (2018). Advanced functional carbons and their hybrid nanoarchitectures towards supercapacitor applications. ChemSusChem.

[40] Samy M.M., Mekhemer I.M., Mohamed M.G., Elsayed M.H., Lin K.-H., Chen Y.-K., Wu T.-L., Chou H.-H., Kuo S.-W. (2022). Conjugated microporous polymers incorporating Thiazolo [5, 4-d] thiazole moieties for Sunlight-Driven hydrogen production from water. Chem. Eng. J..

[41] Zeng W., Zhang Y., Zhao X., Qin M., Li X., Jin W., Zhang D. (2019). One-pot synthesis of conjugated microporous polymers based on extended molecular graphenes for hydrogen storage. Polymers.

[42] Mohamed M.G., Mansoure T.H., Takashi Y., Samy M.M., Chen T., Kuo S.-W. (2021). Ultrastable porous organic/inorganic polymers based on polyhedral oligomeric silsesquioxane (POSS) hybrids exhibiting high performance for thermal property and energy storage. Microporous Mesoporous Mater..

[43] Liao Y., Wang H., Zhu M., Thomas A. (2018). Efficient supercapacitor energy storage using conjugated microporous polymer networks synthesized from Buchwald–Hartwig coupling. Adv. Mater..

[44] Machado T.F., Serra M.E.S., Murtinho D., Valente A.J.M., Naushad M. (2021). Covalent Organic Frameworks: Synthesis, Properties and Applications—An Overview. Polymers.

[45] Zhang S., Yang Q., Wang C., Luo X., Kim J., Wang Z., Yamauchi Y. (2018). Porous Organic Frameworks: Advanced Materials in Analytical Chemistry. Adv. Sci..

[46] Mohamed M.G., Samy M.M., Mansoure T.H., Sharma S.U., Tsai M.-S., Chen J.-H., Lee J.-T., Kuo S.-W. (2022). Dispersions of 1, 3, 4-oxadiazole-linked conjugated microporous polymers with carbon nanotubes as a high-performance electrode for supercapacitors. ACS Appl. Energy Mater..

[47] Fischer S., Schimanowitz A., Dawson R., Senkovska I., Kaskel S., Thomas A. (2014). Cationic microporous polymer networks by polymerisation of weakly coordinating cations with CO_2_-storage ability. J. Mater. Chem. A.

[48] Wang J., Wang L., Wang Y., Zhang D., Xiao Q., Huang J., Liu Y.-N. (2022). Recent progress in porous organic polymers and their application for CO_2_ capture. Chin. J. Chem. Eng..

[49] Mohamed M.G., Tsai M.-Y., Wang C.-F., Huang C.-F., Danko M., Dai L., Chen T., Kuo S.-W. (2021). Multifunctional polyhedral oligomeric silsesquioxane (POSS) based hybrid porous materials for CO_2_ uptake and iodine adsorption. Polymers.

[50] Chen J., Jiang L., Wang W., Shen Z., Liu S., Li X., Wang Y. (2022). Constructing highly porous carbon materials from porous organic polymers for superior CO_2_ adsorption and separation. J. Colloid Interface Sci..

[51] Ibrahim M., Tashkandi N., Hadjichristidis N., Alkayal N.S. (2022). Synthesis of Naphthalene-Based Polyaminal-Linked Porous Polymers for Highly Effective Uptake of CO_2_ and Heavy Metals. Polymers.

[52] Shao B., Zhang Y., Sun Z., Li J., Gao Z., Xie Z., Hu J., Liu H. (2022). CO_2_ capture and in-situ conversion: Recent progresses and perspectives. Green Chem. Eng..

[53] Hanifa M., Agarwal R., Sharma U., Thapliyal P., Singh L. (2023). A review on CO_2_ capture and sequestration in the construction industry: Emerging approaches and commercialised technologies. J. CO2 Util..

[54] Chen X., Lin J., Wang H., Yang Y., Wang C., Sun Q., Shen X., Li Y. (2023). Epoxy-functionalized polyethyleneimine modified epichlorohydrin-cross-linked cellulose aerogel as adsorbents for carbon dioxide capture. Carbohydr. Polym..

[55] Ding M., Liu X., Ma P., Yao J. (2022). Porous materials for capture and catalytic conversion of CO_2_ at low concentration. Coord. Chem. Rev..

[56] Liu J., Wei D., Wu L., Yang H., Song X. (2022). Synergy and heterogeneity of driving factors of carbon emissions in China’s energy-intensive industries. Ecol. Indic..

[57] Mohamed M.G., Chang W.-C., Kuo S.-W. (2022). Crown Ether-and Benzoxazine-Linked Porous Organic Polymers Displaying Enhanced Metal Ion and CO_2_ Capture through Solid-State Chemical Transformation. Macromolecules.

[58] Mohamed M.G., Chen T.-C., Kuo S.-W. (2021). Solid-state chemical transformations to enhance gas capture in benzoxazine-linked conjugated microporous polymers. Macromolecules.

[59] Ejaz M., Samy M.M., Ye Y., Kuo S.-W., Mohamed M.G. (2023). Design Hybrid Porous Organic/Inorganic Polymers Containing Polyhedral Oligomeric Silsesquioxane/Pyrene/Anthracene Moieties as a High-Performance Electrode for Supercapacitor. Int. J. Mol. Sci..

[60] Das N., Paul R., Dao D.Q., Chatterjee R., Borah K., Chandra Shit S., Bhaumik A., Mondal J. (2022). Nanospace Engineering of Triazine–Thiophene-Intertwined Porous-Organic-Polymers via Molecular Expansion in Tweaking CO_2_ Capture. ACS Appl. Nano Mater..

[61] Mohamed M.G., Ahmed M.M., Du W.-T., Kuo S.-W. (2021). Meso/microporous carbons from conjugated hyper-crosslinked polymers based on tetraphenylethene for high-performance CO_2_ capture and supercapacitor. Molecules.

[62] Sen S., Al-Sayah M.H., Mohammed M.S., Abu-Abdoun I.I., El-Kadri O.M. (2020). Multifunctional nitrogen-rich aminal-linked luminescent porous organic polymers for iodine enrichment and selective detection of Fe^3+^ ions. J. Mater. Sci..

[63] Sabri M.A., Al-Sayah M.H., Sen S., Ibrahim T.H., El-Kadri O.M. (2020). Fluorescent aminal linked porous organic polymer for reversible iodine capture and sensing. Sci. Rep..

[64] Wu J.-Y., Mohamed M.G., Kuo S.-W. (2017). Directly synthesized nitrogen-doped microporous carbons from polybenzoxazine resins for carbon dioxide capture. Polym. Chem..

[65] Yu Q., Bai J., Huang J., Demir M., Altay B.N., Hu X., Wang L. (2022). One-Pot Synthesis of N-Rich Porous Carbon for Efficient CO_2_ Adsorption Performance. Molecules.

[66] Mehra P., Paul A. (2022). Decoding Carbon-Based Materials’ Properties for High CO_2_ Capture and Selectivity. ACS Omega.

[67] Yu Q., Bai J., Huang J., Demir M., Farghaly A.A., Aghamohammadi P., Hu X., Wang L. (2023). One-Pot Synthesis of Melamine Formaldehyde Resin-Derived N-Doped Porous Carbon for CO_2_ Capture Application. Molecules.

[68] Weng T.-H., Mohamed M.G., Sharma S.U., Chaganti S.V., Samy M.M., Lee J.-T., Kuo S.-W. (2022). Ultrastable three-dimensional triptycene-and tetraphenylethene-conjugated microporous polymers for energy storage. ACS Appl. Energy Mater..

[69] Ahmed M.M., Imae T., Hill J.P., Yamauchi Y., Ariga K., Shrestha L.K. (2018). Defect-free exfoliation of graphene at ultra-high temperature. Colloids Surf. A Physicochem. Eng. Asp..

[70] Lin Y., Chen Z., Yu C., Zhong W. (2019). Heteroatom-doped sheet-like and hierarchical porous carbon based on natural biomass small molecule peach gum for high-performance supercapacitors. ACS Sustain. Chem. Eng..

[71] Quan T., Goubard-Bretesché N., Härk E., Kochovski Z., Mei S., Pinna N., Ballauff M., Lu Y. (2019). Highly dispersible hexagonal carbon–MoS_2_–carbon nanoplates with hollow sandwich structures for supercapacitors. Chem. Eur. J..

[72] Peng Z., Zou Y., Xu S., Zhong W., Yang W. (2018). High-performance biomass-based flexible solid-state supercapacitor constructed of pressure-sensitive lignin-based and cellulose hydrogels. ACS Appl. Mater. Interfaces.

[73] Bairi P., Shrestha R.G., Hill J.P., Nishimura T., Ariga K., Shrestha L.K. (2016). Mesoporous graphitic carbon microtubes derived from fullerene C 70 tubes as a high performance electrode material for advanced supercapacitors. J. Mater. Chem. A..

[74] Saha D., Li Y., Bi Z., Chen J., Keum J.K., Hensley D.K., Grappe H.A., Meyer III H.M., Dai S., Paranthaman M.P. (2014). Studies on supercapacitor electrode material from activated lignin-derived mesoporous carbon. Langmuir.

[75] Goldfarb J.L., Dou G., Salari M., Grinstaff M.W. (2017). Biomass-based fuels and activated carbon electrode materials: An integrated approach to green energy systems. ACS Sustain. Chem. Eng..

[76] Liu X., Zhou L., Zhao Y., Bian L., Feng X., Pu Q. (2013). Hollow, spherical nitrogen-rich porous carbon shells obtained from a porous organic framework for the supercapacitor. ACS Appl. Mater. Interfaces.

[77] Stimpfling T., Leroux F. (2010). Supercapacitor-type behavior of carbon composite and replica obtained from hybrid layered double hydroxide active container. Chem. Mater..

[78] Oh J.Y., Jung Y., Cho Y.S., Choi J., Youk J.H., Fechler N., Yang S.J., Park C.R. (2017). Metal–Phenolic Carbon Nanocomposites for Robust and Flexible Energy-Storage Devices. ChemSusChem.

[79] Cao J., Jafta C.J., Gong J., Ran Q., Lin X., Félix R., Wilks R.G., Bär M., Yuan J., Ballauff M. (2016). Synthesis of dispersible mesoporous nitrogen-doped hollow carbon nanoplates with uniform hexagonal morphologies for supercapacitors. ACS Appl. Mater. Interfaces.

[80] Kim M., Lim H., Xu X., Hossain M.S.A., Na J., Awaludin N.N., Shah J., Shrestha L.K., Ariga K., Nanjundan A.K. (2021). Sorghum biomass-derived porous carbon electrodes for capacitive deionization and energy storage. Microporous Mesoporous Mater..

