# Supplementary material for: Carbonized Aminal-Linked Porous Organic Polymers Containing Pyrene and Triazine Units for Gas Uptake and Energy Storage
Source: Polymers (Basel). 2023 Apr 14;15(8):1891. doi: 10.3390/polym15081891 (PMC10146094; doi:10.3390/polym15081891)
Supplement: Supplementary file 1 [file polymers-15-01891-s001.zip › polymers-2284852-supplementary.pdf]

Supporting Information

Characterization

FTIR spectra were collected on a Bruker Tensor 27 FTIR spectrophotometer with a resolution of 4 cm^{-1} by using KBr disk method. ^{13}C nuclear magnetic resonance (NMR) spectra were examined by using an INOVA 500 instrument with DMSO as the solvent and TMS as the external standard. Chemical shifts are reported in parts per million (ppm). The thermal stabilities of the samples were performed by using a TG Q-50 thermogravimetric analyzer under a N_2 atmosphere; the cured sample (ca. 5 mg) was put in a Pt cell with heating rate of 20 °C min^{-1} from 100 to 800 °C under a N_2 flow rate of 60 mL min^{-1}. Wide-angle X-ray diffraction (WAXD) patterns were measured by the wiggler beamline BL17A1 of the National Synchrotron Radiation Research Center (NSRRC), Taiwan. A triangular bent Si (111) single crystal was used to get a monochromated beam having a wavelength (λ) of 1.33 Å. The morphologies of the polymer samples were examined by Field emission scanning electron microscopy (FE-SEM; JEOL JSM7610F) and also by transmission electron microscope (TEM) using a JEOL-2100 instrument at an accelerating voltage of 200 kV. BET surface area and porosimetry measurements of samples (ca. 40–100 mg) were measured using BEL Master^{TM}/BEL sim^{TM} (v. 3.0.0). N_2 adsorption and desorption isotherms were generated through incremental exposure to ultrahigh-purity N_2 (up to ca. 1 atm) in a liquid N_2 (77 K) bath. Surface parameters were calculated using BET adsorption models in the instrument's software. The pore size of the prepared samples was determined by using nonlocal density functional theory (NLDFT).

Electrochemical Analysis

Working Electrode Cleaning: Prior to using, the glassy carbon electrode (GCE) was polished several times with 0.05-μm alumina powder, washed with EtOH after each polishing step, cleaned through sonication (5 min) in a water bath, washed with EtOH, and then dried in the oven at 50 °C.

Electrochemical Characterization: The electrochemical experiments were performed in a three-electrode cell using an Autolab potentiostat (PGSTAT204) and 1 M KOH as the aqueous electrolyte. The GCE was used as the working electrode (diameter: 5.61 mm; 0.2475 cm^2); a Pt wire was used as the counter electrode; Hg/HgO (RE-1B, BAS) was the reference electrode. All reported potentials refer to the Hg/HgO potential. A slurry was prepared by dispersing Py-PDT POP and Py-PDT POP-600 (45 wt %), carbon black (45 wt %), and Nafion (10 wt%) in a mixture of (EtOH/ H_2O) (200 μL: 800 μL) and then sonicating for 1 h. A portion of this slurry (10 μL) was pipetted onto the tip of the electrode, which was then dried in air for 30 min prior to use. The electrochemical performance was studied through CV at various sweep rates (5–200 mV s^{-1}) and through the GCD method in the potential range from 0 to -1.00 V (vs. Hg/HgO) at various current densities (0.5–20 A g^{-1}) in 1 M KOH as the aqueous electrolyte solution.

The specific capacitance was calculated from the GCD data using the equation.

$$C_s = (I\Delta t)/(m\Delta V) \qquad \textbf{(S1)}$$

Where C_s (F g^{-1}) is the specific capacitance of the supercapacitor, I (A) is the discharge current, ΔV (V) is the potential window, Δt (s) is the discharge time, and m (g) is the mass of the NPC on the electrode. The energy density (E, W h kg^{-1}) and power density (P, W kg^{-1}) were calculated using the equations.

$$E = 1000C(\Delta V)^2/(2 \times 3600) \quad \textbf{(S2)}$$

$$P = E/(t/3600) \quad \textbf{(S3)}$$

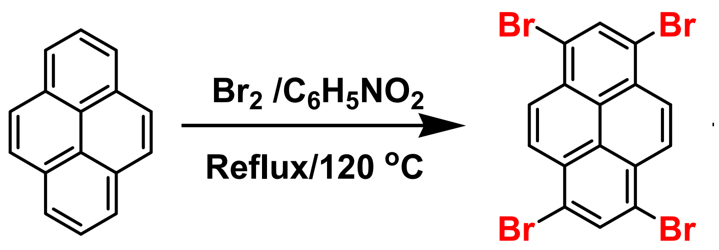

Scheme S1. Synthesis of Py-Br_4.

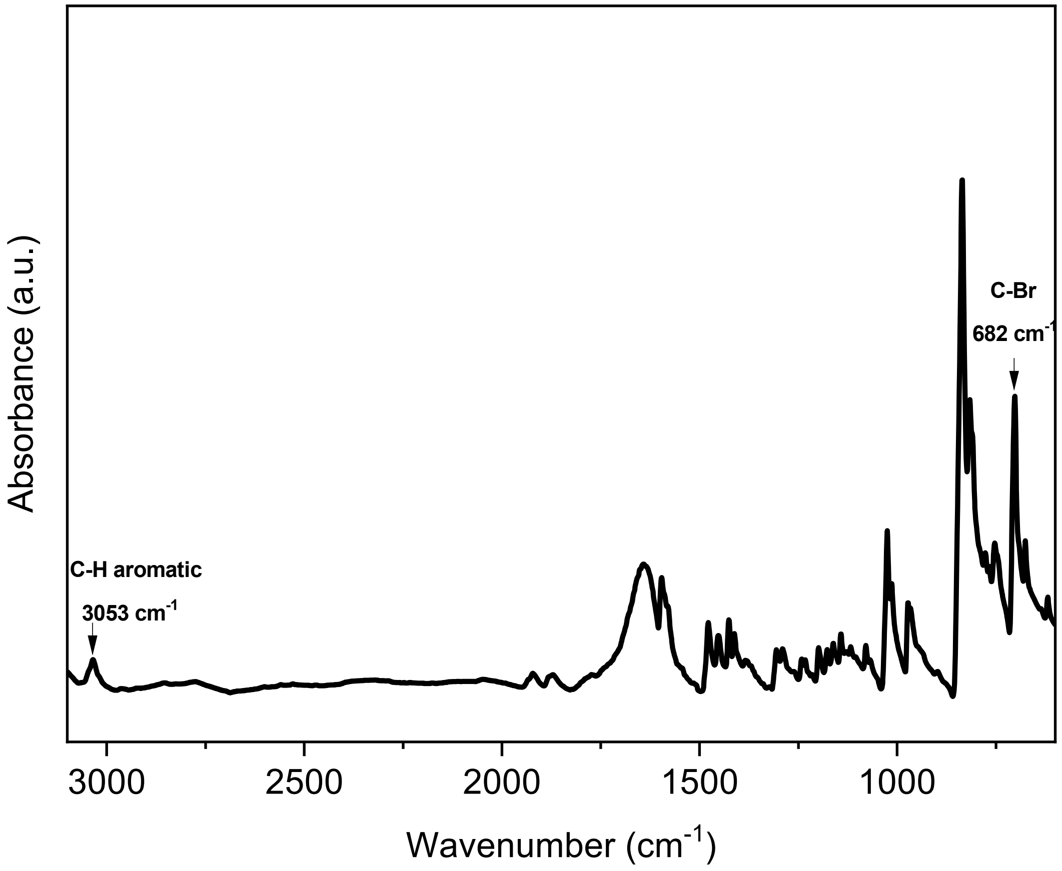

Figure S1. FT-IR spectrum of Py-Br_4.

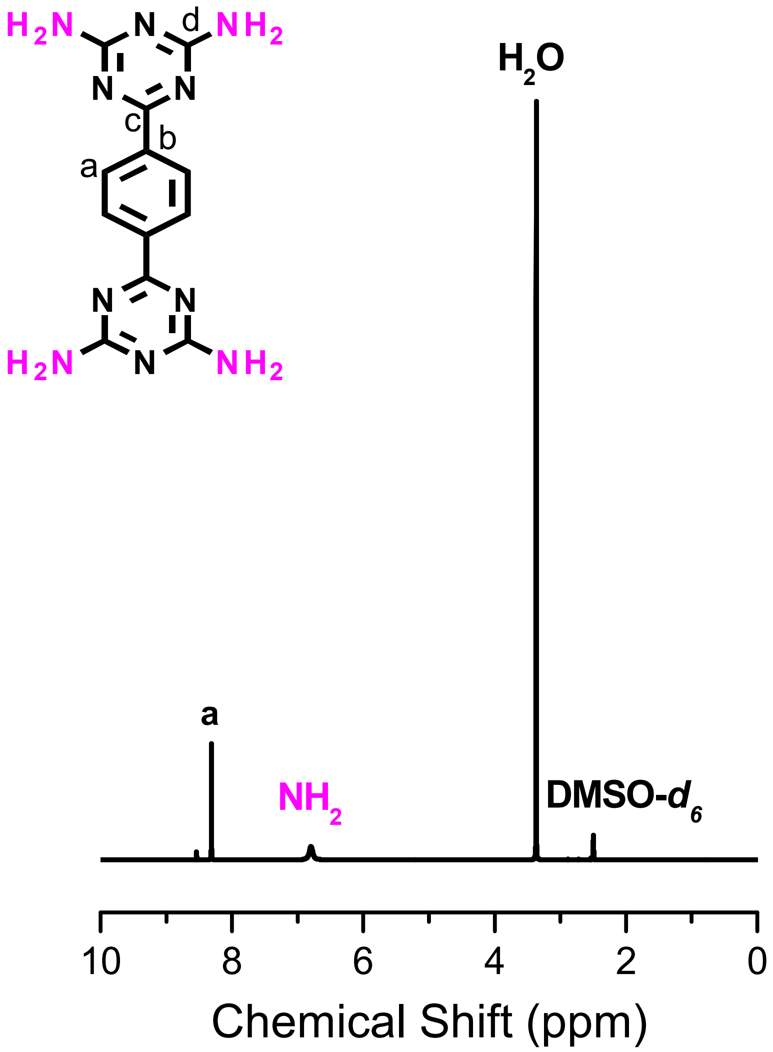

Figure S2. ^{1}H-NMR profile of PDT-4NH2.

Table S1. Comparison between the capacity values of Py-PDT POP-600 with different reported data of three electrode supercapacitor materials [**61,69-80**].

Material	Surface area (m^2 g^{-1})	Capacitance	Reference
Py-PDT POP-600	314	550 F g^{-1}/0.5 A g^{-1}	This work
Py-PDT POP	76	28 F g^{-1}/0.5 A g^{-1}	This work
Heating exfoliated GIC at 2000 °C	50	90 F g^{-1}/5mV s^{-1}	69
Carbons derived from Peach Gum	95	199 F g^{-1}/0.2 A g^{-1}	70
Hollow carbon-MoS_2-carbon nanoplates	543	178 F g^{-1}/1.0 A g^{-1}	71
Lignin-Based and Cellulose Hydrogels	713	292 F g^{-1}/0.5 A g^{-1}	72
Mesoporous graphitic carbon microtubes derived from fullerene C_{70} through heating at 2000 °C	609	212.2 F g^{-1}/5 mV s^{-1}	73
TPE-CPOP1-800	1177	453 F g^{-1}/5 mV s^{-1}	61
TPE-CPOP2-800	1165	200 F g^{-1}/5 mV s^{-1}	61
cross-linked lignin gel impregnated with a surfactant	1148	100 F g^{-1}/5 mV s^{-1}	74
Pyrolysis of pistachio nutshell biomass	1900	45 F g^{-1}/1 mV s^{-1}	75
NPC800	525	230 F g^{-1}/0.5 mV s^{-1}	76
Carbon Composite and Replica Obtained from Hybrid Layered Double Hydroxide Active Container	1535	92.6 μF cm^{-2}	77
tannic acid (TA) and carbon nanotubes (CNTs)	-	147.4 F g^{-1}/0.5 A g^{-1}	78
Mesoporous Nitrogen-Doped Hollow Carbon Nanoplates with Uniform Hexagonal Morphologies	460	95 F g^{-1}/1.0 A g^{-1}	79
Sorghum biomass-derived porous carbon	1674	228 F g^{-1}/5 mV s^{-1}	80
